# An Automated Method for Identifying Artifact in Independent Component Analysis of Resting-State fMRI

**DOI:** 10.3389/fnhum.2013.00343

**Published:** 2013-07-10

**Authors:** Kaushik Bhaganagarapu, Graeme D. Jackson, David F. Abbott

**Affiliations:** ^1^The Florey Institute of Neuroscience and Mental Health, The University of Melbourne, Austin Hospital, Melbourne, VIC, Australia; ^2^Department of Medicine, The University of Melbourne, Melbourne, VIC, Australia; ^3^Department of Radiology, The University of Melbourne, Melbourne, VIC, Australia

**Keywords:** functional magnetic resonance imaging, fMRI, independent component analysis, ICA, automated classification, automatic, artifacts, independent component labeling

## Abstract

An enduring issue with data-driven analysis and filtering methods is the interpretation of results. To assist, we present an automatic method for identification of artifact in independent components (ICs) derived from functional MRI (fMRI). The method was designed with the following features: does not require temporal information about an fMRI paradigm; does not require the user to train the algorithm; requires only the fMRI images (additional acquisition of anatomical imaging not required); is able to identify a high proportion of artifact-related ICs without removing components that are likely to be of neuronal origin; can be applied to resting-state fMRI; is automated, requiring minimal or no human intervention. We applied the method to a MELODIC probabilistic ICA of resting-state functional connectivity data acquired in 50 healthy control subjects, and compared the results to a blinded expert manual classification. The method identified between 26 and 72% of the components as artifact (mean 55%). About 0.3% of components identified as artifact were discordant with the manual classification; retrospective examination of these ICs suggested the automated method had correctly identified these as artifact. We have developed an effective automated method which removes a substantial number of unwanted noisy components in ICA analyses of resting-state fMRI data. Source code of our implementation of the method is available.

## 1 Introduction

Functional magnetic resonance imaging (fMRI) is a non-invasive technique that uses the blood oxygen level dependent (BOLD) effect to explore neural activity (Ogawa et al., 1990). However, BOLD fMRI suffers from numerous sources of structured noise (Biswal et al., 1996; Friston et al., 1996; Glover et al., 2000) which compromises the fMRI signal. These include rapid and slow head movements, physiological activity (breathing and heart-beat), and potential acquisition artifacts. Even after traditional pre-processing steps, such as slice-timing correction, motion correction, high-pass filtering, and spatial smoothing, some of these artifacts still remain (Grootoonk et al., 2000; Lund et al., 2006). To overcome this, the use of data-driven techniques are increasingly being employed to generate potentially valuable information on the nature of signal and noise in fMRI data. In particular, spatial Independent Component Analysis (ICA), has been proposed (McKeown et al., 1998). Spatial ICA is a blind source separation (BSS) technique, that decomposes fMRI data into components which are maximally independent (Hyvärinen, 1999). Each Independent Component (IC) contains a 3D spatial map and a 1D time-course. When compared to traditional fMRI analysis approaches, where a design paradigm and assumptions about the hemodynamic processes in the brain are required to obtain spatial activation maps (Buxton et al., 2004), ICA offers a hypothesis free model to gain further insights in identifying the spatial location of brain activity. However, such an approach, due to its hypothesis free nature begs the question of interpretation of the results. In particular, how does one distinguish between ICs which are signal (i.e., components of neuronal origin) and noise (i.e., due to movement, cardiac pulsations etc.)? Typically, this has been done by visually inspecting each IC and manually categorizing them (McKeown et al., 1998; Moritz et al., 2003; Kelly Jr. et al., 2010). This is however, a very time consuming and subjective procedure which is dependent on the experience of the researcher. For example, Kelly Jr. et al. (2010) provide a detailed description of the criteria to manually classify ICs via visual inspection. They estimate approximately 37 min for classifying 100 ICs which can be a typical yield from lengthy resting-state ICA (Rodionov et al., 2007; LeVan et al., 2010).

Other methods have classified ICs by using paradigm information (Thomas et al., 2002; Calhoun et al., 2005; Kochiyama et al., 2005). Specifically, Calhoun et al. (2005) present an approach for semi-blind ICA analysis of event-related fMRI data by imposing regularization on certain estimated time courses using the paradigm information. This approach, however, is limited to studies where temporal information is available. In some applications it may not be desirable to use temporal information in the classifier. Resting-state functional connectivity is one such application. Another, which is a particular interest of ours, is the data-driven exploration of fMRI prior to an epileptic seizure (Federico et al., 2005), where we have no prior model of expected signal change or event timing (apart from the seizure itself at which point the data acquisition usually ends).

More recently, several automatic techniques have been developed to assist in classifying ICs into categories of noise and signal (Perlbarg et al., 2007; Stevens et al., 2007; Calhoun et al., 2008; Sui et al., 2009; Kundu et al., 2012). Perlbarg et al. (2007) uses both spatial and temporal patterns to categorize ICs into noise and signal. However, their automatic classifier, CORSICA, is limited to identifying physiological noise. Calhoun et al. (2008) utilize a brain atlas to aid sorting ICs. However, atlas based sorting requires strong *a priori* assumptions on the spatial layout of the activation which is not always available. Sui et al. (2009) employ spatial only criterion to automatically classify ICs as they use contrast images that contain no time-domain information. Their method relies on generating cerebrospinal fluid (CSF) red and gray-matter (GM) masks. It can be difficult to obtain an accurate GM mask with fMRI images, especially at higher magnetic field strengths (e.g., 3 T) where image distortions and signal dropout can result in blurred boundaries between gray matter and white matter. Kundu et al. (2012) differentiate BOLD-like functional network components from non-BOLD-like components related to motion, pulsatility, and other nuisance effects based on TE-dependence. While this was found to be a robust method compared to conventional techniques for classifying artifacts, the technique requires a multi-echo acquisition sequence and cannot be applied to conventional single-echo fMRI data.

Other automatic techniques based on machine learning algorithms have been applied to identify artifactual ICs (De Martino et al., 2007; Tohka et al., 2008). De Martino et al. (2007) represents each IC in a multidimensional space, called an IC-fingerprint. Using these IC-fingerprints, they classify ICs into various categories of signal and noise. Tohka et al. (2008) uses a combination of spatial and temporal criteria to aid in classifying signal and noise via global decision trees. However, their classifier overlooks physiological noise. Moreover these two techniques are primarily dependent on a training data set.

We sought to overcome some of the limitations of existing classifiers by developing an artifact identification method that:

- Does not require temporal information about the fMRI paradigm.
- Does not require the user to train the algorithm.
- Requires only the EPI images (additional acquisition of anatomical images is not required).
- Is able to identify a high proportion of artifact-related ICs without removing components that are likely to be of neuronal origin.
- Can be applied to resting-state fMRI.
- Is automated, requiring minimal or no human intervention.

We are not aware of any existing IC artifact identification method that contains all of the above features. We have dubbed our method the Spatially Organized Component Klassifikator (SOCK). In the context of this paper, we mean by “Klassifikator” (a German word meaning classifier) the ability to distinguish between ICs dominated by artifact and those containing possible neuronal signal. We note from the outset that our approach is designed to complement rather than replace existing approaches. A limitation in some applications can be a strength in others. We designed SOCK for particular applications where the features listed above are the highest priorities.

## 2 Methods

### 2.1 Methods overview

**Figure 1:**
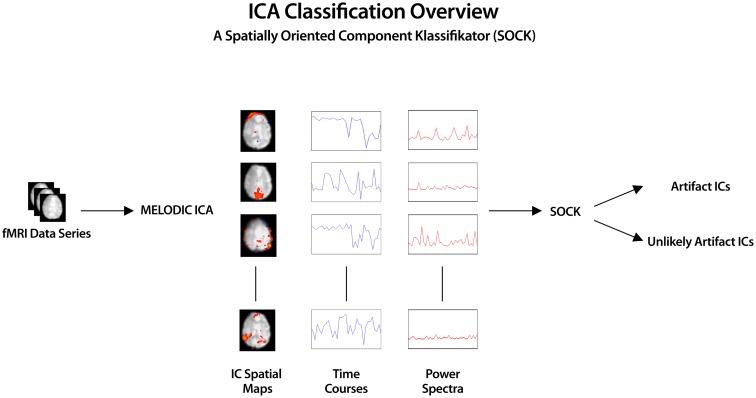
**ICA was applied to pre-processed fMRI data yielding spatial component maps with associated time courses and power spectra**. SOCK automatically distinguishes between ICs dominated by artifact (Artifact category) and those containing possible neuronal signal (Unlikely Artifact category) by calculating IC features (smoothness measure, edge, CSF, and temporal frequency power).

The overview of the automatic IC classification process is given below (see also Figure 1).

1. ICA was applied to the pre-processed fMRI data (see Section 2.5) using MELODIC (Beckmann and Smith, 2004), yielding both thresholded (*P* < 0.05) and unthresholded ICs and associated time courses and power spectra^1^.
2. Calculation of features (smoothness measure, edge, CSF, and temporal frequency power) for each IC was computed via the SOCK algorithm.
3. Based on the above features, ICs dominated by artifact are classified into an Artifact category and all other ICs (i.e., those containing possible neuronal signal) into an Unlikely Artifact category.

Source code of our implementation of the method is available at http://www.brain.org.au/software.

### 2.2 ICA decomposition

The idea behind ICA is to decompose the 4D fMRI time series into a linear combination of spatially independent component maps with an associated time-course (McKeown et al., 1998; Hyvärinen, 1999). This is expressed mathematically as follows:

(1) $$X=\sum_{i=1}^{N}T_{i}S_{i}$$

where *X* is a *K* × *M* matrix (*K* = number of samples and *M* = number of time courses) of the fMRI time series, *S* the *N* × *M* matrix whose rows *S_i_* (*i* = 1, …, *N*) represent the *i^th^* spatial component (*K* ≤ *T*) and *T* is the *K* × *N* mixing matrix (unknown), whose columns *T_i_* (*i* = 1, …, *N*) contain the time courses of the *N* sources. Estimating the number of sources, *N* is done in the pre-processing step, usually via PCA (Beckmann and Smith, 2004).

The only constraint enforced in this decomposition is that each of the component maps, *S_i_*’s are spatially independent. This is equivalent to saying that all *S_i_*’s, with the exception of one have to be non-Gaussian. Structured non-Gaussian noise (head motion and physiological noise) in the fMRI data series is not explicitly modeled, but is treated as an independent source in the ICA decomposition (McKeown et al., 1998; Hyvärinen, 1999).

The ICA decomposition is done by estimating the mixing matrix, *T*, by minimizing redundancy in the spatial maps of the components, *S*. This can be mathematically expressed as:

(2) $$S=\sum_{i=1}^{N}W_{i}X_{i}$$

where matrix *W*, called the “un-mixing” matrix, is the inverse of *T*. Several freely available software packages are available to perform this decomposition; we used MELODIC which is part of the FSL package (Beckmann and Smith, 2004). The output is a set of spatial maps (*S_i_*) with associated time courses (*T_i_*) and power spectra (*PS_i_*). These then form the input for the automatic classifier, SOCK.

### 2.3 Calculation of IC features

SOCK automatically identifies artifact in each IC using features likely to indicate motion, physiological noise, or machine or undetermined noise. To achieve this, each IC is assessed for the presence of substantial edge-only activity, activity in the ventricles, or a large number of isolated very small clusters or isolated voxels (i.e., a “spotty” appearance), respectively. Specifically, we use four measures:

1. Smoothness measure. This assesses the contributions of low and high *spatial* frequency content for each IC (Section 2.3.1).
2. Edge activity measure. This assesses the extent of activity in peripheral areas of the brain, via an edge mask (Section 2.3.2).
3. CSF activity measure. This assesses the extent of activity in ventricular areas of the brain, via a CSF mask (Section 2.3.3).
4. Temporal Frequency Noise (TFN) measure. This assesses the power in *temporal* frequency beyond 0.08 Hz (Section 2.3.4).

#### 2.3.1 Smoothness measure

The spotty appearance of an IC, which reflects the degree of smoothness, is identified by observing spatial frequencies via a Fourier Transform. We assume components that are likely to be of neuronal origin will be relatively smooth and that by observing contributions in spatial frequency, we can distinguish between ICs that are smooth and unsmooth. The framework for the smoothness criterion is as follows.

Let *F*^i^(*K*_*x*_, *K*_*y*_, *K*_*z*_) be the 3D Discrete Fourier Transform of IC, *i*. That is,

(3) $$\begin{array}{l} F^{i}\left(K_{x},K_{y},K_{z}\right)=\sum_{X}\sum_{Y}\sum_{Z}S^{i}\\ \;\left(X,Y,Z\right)e^{-2\pi j\left(K_{x}.X.X_{0}+K_{y}.Y.Y_{0}+K_{Z}.Z.Z_{0}\right)} \end{array}$$

where *j* is an imaginary unit, *X*, *Y*, and *Z* are vectors corresponding to the fMRI image dimensions and *X*_0_, *Y*_0_, and *Z*_0_ are the step sizes between consecutive samples in the *X*, *Y*, and *Z* directions respectively. *K_x_*, *K_y_*, and *K_z_* are vectors in the Fourier space and *S* is the intensity in the image space. A typical Fourier Transform for a *single slice* is illustrated in 2A. While a 3D Discrete Fourier Transform is implemented in SOCK, we show a 2D illustration for simplicity. Data in the center of this figure contains low spatial frequency information about the image, while data near the periphery represents high spatial frequencies. We apply the above Discrete Fourier Transform to *unthresholded* ICs, thus capturing all spatial frequency modes to assess whether or not an IC is smooth. To classify the extent of spatial smoothness of a particular IC, we calculate a ratio of low to high frequency information.

**Figure 2:**
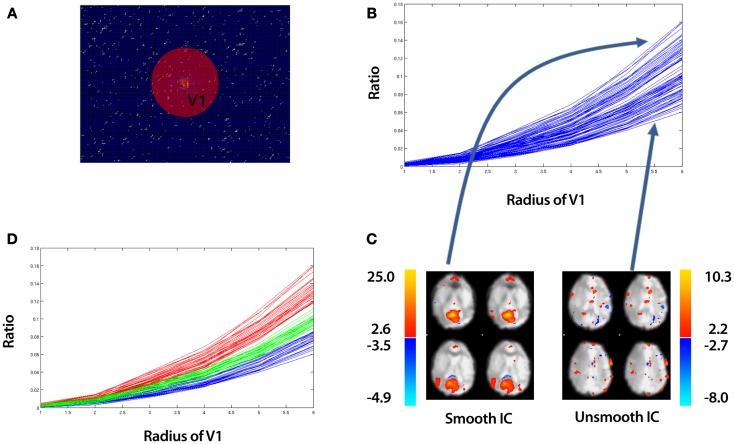
**An overview of identifying “spotty” artifact**. The example data is taken from an ICA of a control that underwent a 10 min resting-state fMRI study (see Section 2.5). **(A)** Discrete Fourier Transform of a *single slice* of an IC. While a 3D Discrete Fourier Transform is implemented in SOCK, we show a 2D illustration for simplicity. Data in the center of figure contains low spatial frequency information about the image, while data near the periphery represents high spatial frequencies. The sphere with volume *V*1 is an arbitrary region which contains low spatial frequency information. **(B)** A plot of the ratio of low to high frequency information [equation (6)] vs. the radius of sphere *V*1 for all ICs. **(C)** A spatial map of the top and bottom curves corresponding to the smoothest and least smooth IC respectively. **(D)** Applying k-means clustering to split the ICs into a set of “smooth” (red curves), “subsmooth” (green curves), and “unsmooth” (blue curves) ICs. Each line in **(B,D)** represents the ratio for a particular IC calculated over all slices, not just the slice shown in **(A)**.

Let *L^i^* be the low frequency information contained within volume, *V*1 (see Figure 2A) for IC, *i*. That is,

(4) $$L^{i}=\underset{V1_{x}}{\sum }\underset{V1_{y}}{\sum }\underset{V1_{z}}{\sum }F^{i}\left(K_{x},K_{y},K_{z}\right)$$

where *V*1*_x_*, *V*1*_y_*, and *V*1*_z_* are vectors corresponding to the dimensions in Fourier space of volume, *V*1. Let *H^i^* be the high frequency information contained outside of volume, *V*1 for IC, *i*. That is,

(5) $$H^{i}=\underset{K_{x}-V1_{x}}{\sum }\underset{K_{y}-V1_{y}}{\sum }\underset{K_{z}-V1_{z}}{\sum }F^{i}\left(K_{x},K_{y},K_{z}\right)$$

We then define, *R^i^* as the ratio of low to high frequency information for IC, *i*. That is,

(6) $$R^{i}=\frac{L^{i}}{H^{i}}$$

This ratio is a function of the radius of the volume, *V*1. As we increase the radius of *V*1, we increase the volume of low intensity frequencies contributing to the ratio. Plotting equation (6) as a function of different radius values, we obtain curves such as the example shown in Figure 2B. Each curve represents a different IC. These are referred to as *ratio curves* from here on in. These *ratio curves* naturally organize themselves from top to bottom representing the smoothest IC at the top to the least smooth IC at the bottom (Figure 2C).

To distinguish between smooth and unsmooth ICs, we apply a k-means clustering in a 2D feature space (Euclidean distance metric) implemented in MATLAB R2010b (The MathWorks Inc., Natick, MA, USA) to the *ratio curves*. Firstly, we split the *ratio curves* into two clusters:

(7) $$(ClusterA,\;ClusterD)=kmeans\left(R^{i},\;2\right)$$

We further split the lower cluster into two clusters:

(8) (ClusterB, ClusterC)=kmeans(ClusterD, 2)

This yields three sets of vectors (*ClusterA*, *ClusterB*, *ClusterC*), which contain *ratio curves*. We label these clusters (*Smooth*, *Subsmooth*, *Unsmooth*). An example of the clustering is shown in Figure 2D.

#### 2.3.2 Edge activity measure

**Figure 3:**
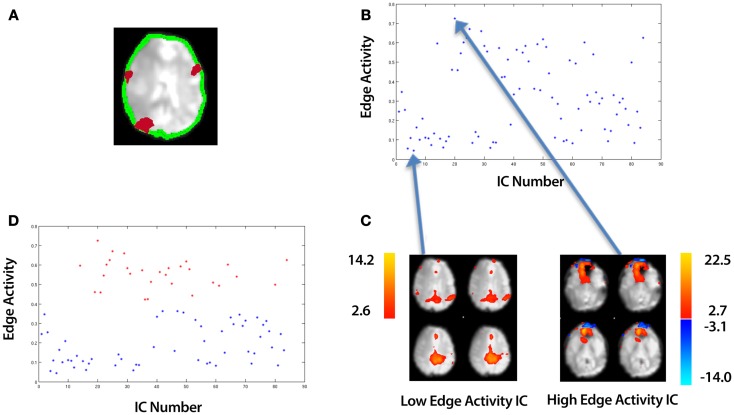
**An overview of identifying motion artifact**. The example data is taken from an ICA of a control that underwent a 10 min resting-state fMRI study (see Section 2.5). **(A)** An edge mask (in green) automatically created by an SPM sub-routine, “New Segment” (single slice shown). This is overlaid onto a mean functional image. In red are an illustration of contiguous clusters which overlap the edge mask. **(B)** A plot of the edge activity for all ICs. **(C)** A spatial map of the top and bottom points corresponding to the highest and lowest edge activity ICs respectively. **(D)** Using k-means clustering, the ICs are automatically divided into a set of “High Edge Activity” components (points colored red in the scatter-plot) and “Low Edge Activity” components (points colored blue in the scatter-plot). Each point in the scatter-plot represents the edge activity for a particular IC summed over all slices.

It has been demonstrated that in ICA of fMRI data, gross subject motion can result in artifactual activity at the edge of the brain (McKeown et al., 1998). We therefore assess the amount of activity within an edge mask, an example of which is shown in Figure 3A (single slice shown). For this we utilize the sub-routine, “New Segment” from the SPM8 software package (The Wellcome Trust Centre for Neuroimaging^2^), applied to the mean functional image. “New Segment” generates two edge masks covering the inner and outer brain boundary; we amalgamate these masks to produce a single edge mask. To identify ICs characterized by gross motion artifact, we define a variable, *edge activity*, which is a measure of the extent of activation overlapping the edge mask. The edge activity, *EA^i^* for each IC, *i* is defined as follows:

(9) $$EA^{i}=\frac{\sum_{k}OE_{k}}{E_{v}}$$

where *E_v_* is the volume of the edge mask and *OE_k_*, ∀*k* = 1, 2, …, *n* is the volume of those contiguous clusters which overlaps the edge mask with there being *n* of these clusters. An illustration of these clusters is shown in Figure 3A for a single slice. We used the “locmax.m” function within the FMRISTAT software (Worsley et al., 2002) to extract these contiguous clusters.

Plotting equation (9) for each IC, *i* produces a plot such as that shown in Figure 3B. Each point represents a different IC with the highest and lowest points corresponding to the ICs with the highest edge activity and lowest edge activity respectively (Figure 3C). Similar to the technique used in clustering the smooth and unsmooth ICs, we employ k-means clustering in a 2D feature space (Euclidean distance metric) to group edge activity into two clusters:

(10) $$(ClusterA,\;ClusterB)=kmeans\left(EA^{i},\;2\right)$$

where (*ClusterA*, *ClusterB*) are two vectors which contain edge activities. We label these clusters (*Low Edge Activity*, *High Edge Activity*). An example of the clustering is shown in Figure 3D. In addition to the adaptive clustering we employ a fixed threshold rejecting ICs independent of any other criteria when they have a 50% or greater volume of activity overlapping the edge mask. This threshold was identified by testing combinations of thresholds on data independent from the data presented here (see Appendix B).

#### 2.3.3 CSF activity measure

**Figure 4:**
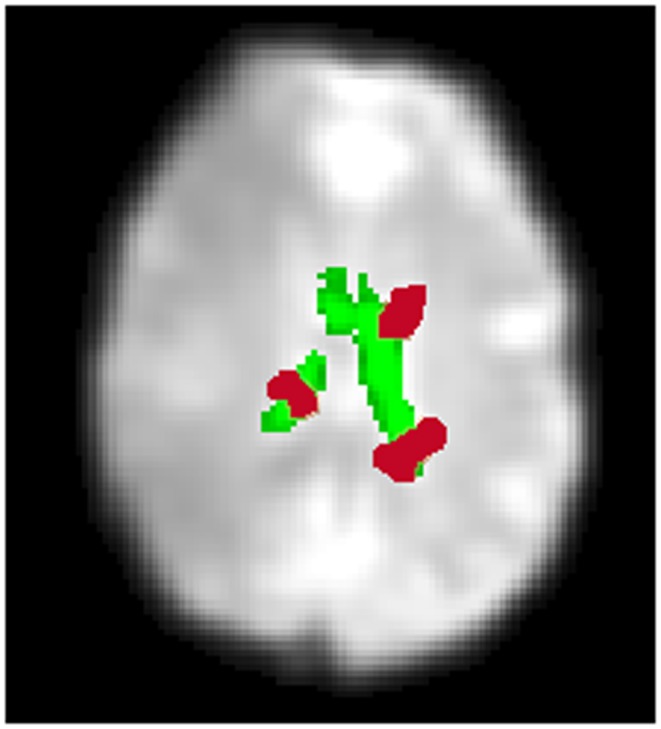
**An overview of identifying CSF artifact**. A CSF mask automatically created by an SPM sub-routine, “New Segment” is shown in green (single slice shown). This is overlaid onto a mean functional image. In red are an illustration of contiguous clusters which overlap the CSF mask. If the volume of activity overlapping the CSF mask is 10% or greater, the IC is labeled *High CSF Activity* (*Low CSF Activity* otherwise). The CSF activity for a particular IC calculated over all slices, not just the slice shown above.

Physiological noise, due to breathing and heart-beat, is often most evident in or at the borders of CSF regions such as the ventricles (Weisskoff et al., 1993; Windischberger et al., 2002). To detect such noise we create a CSF mask, isolating the lateral ventricles. An example is shown in Figure 4 (single slice shown). For this we utilize the sub-routine, “New Segment” from the SPM8 software package [The Wellcome Trust Centre for Neuroimaging (see text footnote 2)], applied to the mean functional image. To isolate the lateral ventricles, we manually defined this region on the Montreal Neurological Institute (MNI) templates included in SPM 8. To identify ICs characterized by CSF artifact, we define a variable, *CSF activity*, which is a measure of the extent of activation overlapping the CSF mask. The CSF activity, *CA^i^* for each IC, *i* is defined as follows:

(11) $$CA^{i}=\frac{\underset{k}{\sum }OC_{k}}{\mathrm{CS}F_{v}}$$

where *OC_k_*, ∀*k* = 1, 2, …, *m* is the volume of the contiguous clusters which overlaps the CSF mask with there being *m* of these clusters. An illustration of these clusters is shown in Figure 4 for a single slice. *CSF_v_* is the volume of the CSF mask.

Unlike the smoothness and motion artifact measures, we do not employ a clustering technique to identify ICs characterized by CSF artifact. Instead, we employ a fixed threshold. If the volume of activity overlapping the CSF mask is 10% or greater, the IC is labeled *High CSF Activity* (*Low CSF Activity* otherwise). In addition, we reject ICs independent of any other criteria when they have a 30% or greater volume of activity overlapping the CSF mask. This threshold was identified by testing combinations of thresholds on data independent from the data presented here (see Appendix B).

#### 2.3.4 Temporal frequency noise measure

The dominant period of the hemodynamic Response Function (HRF) is approximately 12 s (from onset to return to baseline, ignoring the post-stimulus undershoot) (Chapter 10, Huettel et al., 2009). Therefore we expect the dominant frequency for components which exhibit BOLD neuronal signal to be about 1/12 or 0.08 Hz. Hence to identify activity that is unlikely to come from the BOLD HRF, we quantify the temporal power beyond 0.08 Hz.

**Figure 5:**
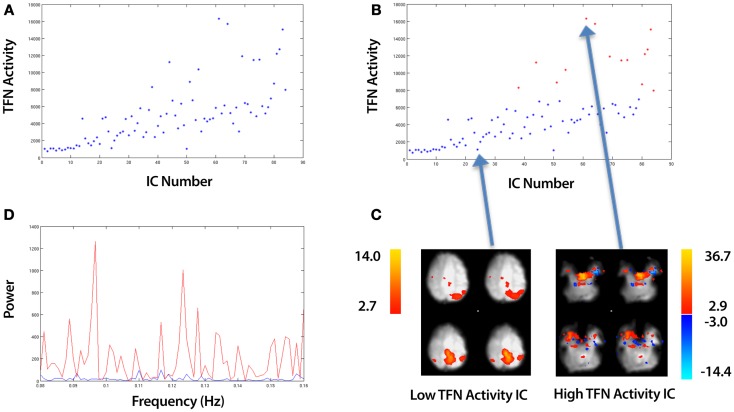
**An overview of identifying ICs with Temporal Frequency Noise (TFN)**. The example data is taken from an ICA of a control that underwent a 10 min resting-state fMRI study (see Section 2.5). **(A)** A plot of the TFN activity (sum of power spectrum values from 0.08 Hz to Nyquist frequency) for all ICs. **(B)** Applying k-means clustering to split the ICs into a set of Low and High TFN ICs. **(C)** A spatial map of the top and bottom points corresponding to the highest and lowest TFN activity ICs respectively. **(D)** The associated power spectra for these ICs with the blue and red curves representing the low and high TFN ICs respectively. This is a zoomed in view showing only frequencies beyond 0.08 Hz which is the region of interest.

To identify ICs characterized by high frequency noise in the time series, we define a variable, Temporal Frequency Noise (TFN), which is a measure of the extent of temporal power beyond 0.08 Hz. The TFN, *TFN^i^* for each IC, *i*, is defined as follows:

(12) $$TFN^{i}=\sum_{0.08}^{f_{Nyquist}}PS^{i}$$

where *PS^i^*, are the power spectrum values for each IC, *i*, which are provided by the MELODIC ICA (Beckmann and Smith, 2004) and *f_Nyquist_* is Nyquist frequency. This formula in essence calculates the sum of all power spectrum values from 0.08 Hz to the Nyquist frequency. Plotting equation (12) for each IC, *i* produces a plot, such as the example shown in Figure 5A. Each point represents a different IC. K-means clustering in a 2D feature space (Euclidean distance metric) is employed to cluster TFN values into two clusters:

(13) $$(ClusterA,\;ClusterB\text{)=}kmeans\left(TFN^{i},\;2\right)$$

where (*ClusterA*, *ClusterB*) are two vectors which contain TFN values. We label these clusters (*High TFN*, *Low TFN*) which correspond to ICs with high and low TFN values respectively. An example of the clustering is shown in Figure 5B, with the spatial maps for the highest and lowest points shown in Figure 5C. The associated power spectra for these ICs are shown in Figure 5D with the blue and red curves representing the low and high TFN ICs respectively. This is a zoomed in view showing only frequencies beyond 0.08 Hz which is the region of interest.

### 2.4 Classification of ICs

Based on the above features, ICs dominated by artifact are identified using the conditions given in Table 1. These were established by the authors based on their experience in visually classifying components from independent data (5 subjects scanned on the same scanner as data sets 1 and 2 in Section 2.5).

**Table 1:** **The conditions used to automatically identify ICs dominated by artifact**.

Smoothness	Edge activity	CSF activity	Temporal frequency noise
Unsmooth	–	–	–

Subsmooth	–	–	High

Smooth	High	High	–

–	High (above 50%)	–	–

–	–	High (above 30%)	–

*Each row indicates a combination of conditions for which the IC is classified as artifact. For example an IC is classified as artifact if it is subsmooth and has high TFN activity*.

### 2.5 fMRI data

We validate SOCK for individual ICA analyses in 50 subjects, from three separate data sets. All were resting-state studies. See Table 2 for a summary.

**Table 2:** **A summary of the three different data sets used to verify SOCK**.

Data set	No. of subjects	Length of study (min)	TR (s)	Pre-processed
1	9	60	3.0	Yes
2	21	10	3.6	Yes
3	20	9	2.0	No

*In total, fMRI data of 50 healthy control subjects were used which had different: study lengths, TR’s, and pre-processing pipelines*.

#### 2.5.1 Data sets 1 and 2

The first two data sets consisted of resting-state data from thirty healthy control subjects that had participated in studies at our institute (Waites et al., 2005, 2006; Lillywhite et al., 2009; Abbott et al., 2010). Ethics approval was obtained from the Austin Health Human Research Ethics Committee or the Howard Florey Institute of Experimental Physiology and Medicine Human Research Ethics Committee and each subject gave informed consent.

Participants were instructed to close their eyes and relax without falling asleep and without focusing on anything in particular. A single run of fMRI data was collected for each of the participants; each run was 60 min in 9 of the participants and 10 min in 21 of the participants.

The fMRI studies were carried out with a 3 T GE Signa LX whole body scanner (General Electric, Milwaukee, WI, USA), using a standard birdcage quadrature head coil. Functional images were acquired as a series of gradient-recalled echo planar imaging (GR-EPI) volumes (TR/TE = 3,000/40 ms in 9 of the participants and TR/TE = 3,600/40 ms in 21 of the participants, 25 oblique slices 4 mm thick + 1-mm gap, voxel size = 1.875 mm × 1.875 mm × 5 mm, 24-cm field of view (FOV), 128 × 128 matrix). The first 14 volumes were discarded (to allow the scanner time to reach steady-state and the subject to become settled to the procedure).

fMRI data were processed using SPM8 software (Wellcome Department of Imaging Neuroscience, London, UK ^3^) with the aid of iBrain^TM^ (Abbott and Jackson, 2001) and the iBrain^TM^ Analysis Toolbox for SPM (Abbott et al., 2011) ^4^. In brief, pre-processing included slice-timing correction, motion correction (realignment), and non-linear warping to a custom local template approximating that of the standard Montreal Neurological Institute (MNI) template supplied with SPM8. The spatially normalized image data were smoothed with an 8 mm isotropic Gaussian kernel and were written at a voxel size of 2 mm × 2 mm × 2 mm. No further pre-processing was carried out in FSL prior to ICA being performed using the MELODIC tool (Beckmann and Smith, 2004).

#### 2.5.2 Data set 3 (functional connectomes data)

The third set of data consisted of resting-state fMRI data with a relatively short TR from twenty healthy controls obtained from the 1000 Functional Connectomes Project website (Biswal et al., 2010, data set 2 from Table S1 with TR = 2,000 ms, 34 slices and voxel size = 3 mm × 3 mm × 3 mm). Participants were instructed to open their eyes without focusing on anything in particular. fMRI data was collected for each of the participants in a 9 min study. The fMRI studies were carried out with a 3 T scanner (make of scanner not specified). Functional images were acquired using a sequential ascending sequence, discarding the first 5 time points of each time series.

No pre-processing [slice-timing correction, motion correction (realignment), normalization, or smoothing] was carried out on this data set.

Preforming ICA using the MELODIC tool, yielded both thresholded and unthresholded ICs and associated time courses and power spectra which were then inputs for SOCK (Figure 1).

In order to evaluate the performance of our algorithm, we assessed SOCK’s classification against manual classification. An expert manual classification of components as either artifact or unlikely artifact was performed blinded to the SOCK classification. The manual classification used visual inspection criteria similar to that outlined in Kelly Jr. et al. (2010) and had previously been applied by consensus of all the authors on data independent from that presented here. In the present study, author KB manually classified all components, and author DA additionally manually classified all ICs in the 1000 Functional Connectomes Project dataset. For all 50 data sets, the number of discordant components [those which were identified as artifact by SOCK but as unlikely artifact by an expert (KB or DA)] were examined in an effort to understand the reason for discordance.

## 3 Results

### 3.1 ICA analysis and SOCK classification

MELODIC ICA was applied to resting-state fMRI data acquired in 50 healthy control subjects across three data sets:

1. Data set 1: 7 male, 2 female; age range 7–11 years, mean = 8.8, SD = 1.6
2. Data set 2: 14 male, 7 female; age range 17–40 years, mean = 24.4, SD = 5.9
3. Data set 3: 20 male; age range 19–38 years, mean = 23.4, SD = 5.3

A total of 2,722 components (average of 54 components per subject) were obtained. SOCK classified between 26 and 72% of each subject’s components as artifact (mean 55%). See Table 3 for a summary. A comprehensive list of the ICA decomposition and the SOCK classification for all 50 subjects is also provided in the Appendix (see Tables A1–A3 in Appendix A).

**Table 3:** **A summary of the SOCK classification for 50 subjects**.

Data set	Number of subjects	SOCK classification			% of rejectedICs	Number of discordant ICs
		Total number of ICA components	Artifact	Unlikely artifact
1	9	758	399	359	53 (39–71)	5
2	21	410	193	217	47 (26–61)	0
3	20	1,554	902	652	58 (42–72)	2

Total	50	2,722	1,494	1,228	55 (26–72)	7

*The last column indicates the components which disagree with an experts classification (see Section 3.2)*.

The time required for SOCK to run, including the automatic generation of the edge and CSF masks, was approximately 2 min per subject on a PC equipped with an Intel Quad-Core i7-2,600 3.4 GHz CPU.

**Figure 6:**
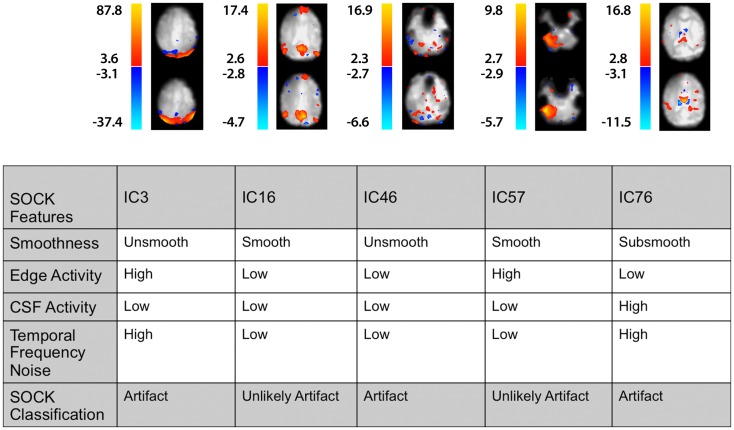
**A selected set of spatial maps from an ICA and corresponding SOCK classification for Subject 4**. The numbering of the ICs is based on the order of extraction in the ICA decomposition.

We show below a case example of each of the SOCK criteria for ICs from a MELODIC ICA on one of the 50 subjects (Table 4; Figure 6). ICA yielded 87 components for this particular subject, out of which 44 (51%) ICs were classified as artifact. No discordant ICs were identified for this particular subject.

**Table 4:** **A summary of the SOCK classification for one (Subject 4) of the 30 subjects**.

Total number of ICA components	SOCK classification		% of rejected ICs	Number of discordant ICs
	
	Artifact	Unlikely artifact		
87	44	43	51	0	

*ICA yielded 87 components for this particular subject, out of which 44 (51%) ICs were classified as artifact. No discordant ICs were identified*.

Figure 6 illustrates the spatial maps and the SOCK classification of a selected set of components from an ICA for this subject. The numbering of the ICs is based on the order of extraction in the ICA decomposition. For example, IC16 has been classified by SOCK as unlikely artifact as it has been clustered into the smooth category and has low edge and CSF activity and low TFN.

### 3.2 Classification performance

We assess the performance of SOCK by calculating the sensitivity, that is, the proportion of components SOCK classifies in the artifact category and the specificity, how many of these components are actually artifact. Table 3 indicates that on average, 55% of ICs were classified in the artifact category. That is, SOCK was able to approximately halve the number of ICs we would otherwise need to look at.

**Figure 7:**
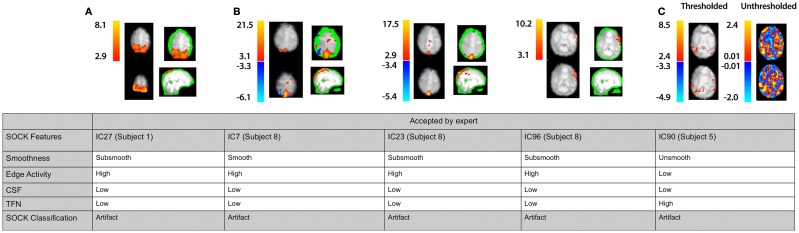
**Spatial maps from an ICA and corresponding SOCK classification for all discordant ICs among the first 30 subjects (data sets 1 and 2)**. All these ICs were classified by an expert as unlikely artifact, whereas SOCK classified them as artifact. The numbering of the ICs is based on the order of extraction in the ICA decomposition. **(A,B)** Subjects 1 and 8: thresholded spatial maps of IC27 for subject 1 and ICs 7, 23, and 96 for subject 8 overlaid on an edge mask (in green, single slice shown) reveals a significant proportion of activation overlapping the edge mask. **(C)** Subject 5: a closer look at the unthresholded spatial map of IC90 (shown on the right of the thresholded map), reveals that the IC is not as smooth as it appears; compared to viewing the thresholded spatial map.

**Figure 8:**
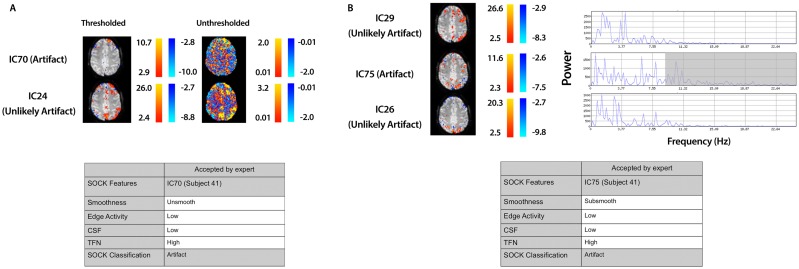
**Spatial maps from an ICA and corresponding SOCK classification for all discordant ICs among the last 20 subjects (data set 3)**. All these ICs were classified by an expert as unlikely artifact, whereas SOCK classified them as artifact. The numbering of the ICs is based on the order of extraction in the ICA decomposition. **(A)** Subject 41, IC70: SOCK rejected this IC as artifact as it was unsmooth. However, an IC with overlapping spatial regions, but with a greater degree of smoothness was observed in the ICA decomposition (compare unthresholded maps of IC70 vs. IC24). IC24 was not classified as artifact by SOCK. **(B)** Subject 41, IC75: SOCK rejected this IC as artifact as it had substantial temporal frequency noise (see shaded region of power spectrum). However, ICs with overlapping spatial regions, but with less temporal frequency noise were observed in the ICA decomposition (compare power spectra of IC75 vs. ICs 26 and 29). These ICs were not classified as artifact by SOCK.

We assessed the specificity by comparing SOCK’s classification against manual classification done by an expert (KB or DA) blinded to the SOCK classification. An expert manually classified each IC into either an artifact or unlikely artifact category. All the ICs which SOCK classified as artifact were compared to ICs which the experts classified. Only 0.3% (7) of components identified as artifact by SOCK were discordant with the manual classification (last column of Table 3); retrospective examination of these ICs suggested SOCK had correctly identified these as artifact. All seven discordant components with their spatial maps and SOCK features are provided in Figures 7 and 8.

#### 3.2.1 IC27 (subject 1)

IC27 in subject 1 (Figure 7A) was accepted by an expert because it contained smooth activity in regions of gray matter and was free from CSF artifact and high TFN. However, SOCK classified this IC as artifact as it contains greater than 50% of volume of activity overlapping the edge mask (see Table 1).

#### 3.2.2 IC7, IC23, and IC96 (subject 5)

IC7, IC23, and IC96 in subject 8 (Figure 7B) were accepted by an expert as they all appeared smooth and did not have gross edge or CSF activity. However, a closer look at the thresholded spatial maps of each IC overlaid on the edge mask (in green) reveals that a significant proportion of activation is overlapping the edge mask. Both axial and coronal views are shown to clearly indicate this. Hence, SOCK classified these ICs as artifact as they contain greater than 50% of volume of activity overlapping the edge mask (see Table 1).

#### 3.2.3 IC90 (subject 5)

IC90 in subject 5 (Figure 7C) was accepted by an expert as it was smooth and did not have gross edge or CSF activity. However, a closer look at the unthresholded spatial map of IC90 (shown on the right of the thresholded map) reveals that the IC is not as smooth as it appears compared to viewing the thresholded spatial map. Hence, SOCK clustered it in the unsmooth category and subsequently classified it as artifact (see Table 1).

#### 3.2.4 IC70 and IC75 (subject 41)

IC70 and IC75 in subject 41 (Figure 8) were not rejected by an expert as they appeared to contain some possible neuronal activity. However these components were rejected by SOCK. SOCK determined IC70 was not sufficiently spatially smooth. Retrospective examination of the ICA decomposition revealed another IC (IC24, not rejected by SOCK) that had overlapping spatial regions (Figure 8A). Examination of the unthresholded component maps (not considered during manual classification) revealed the rejected component did indeed have a less smooth spatial pattern than the accepted component (Figure 8A). IC 75 was rejected by SOCK due to substantial temporal frequency noise. In this case, retrospective examination revealed two ICs (26 and 29) with spatial maps overlapping the apparent neuronal activity in the rejected component. These other components had less temporal frequency noise (as can seen in the shaded area of the power spectrum, Figure 8B) and were not rejected by SOCK.

## 4 Discussion

We have illustrated a general approach for the identification of artifact in independent components derived from fMRI primarily using spatial criteria. The motivation for our algorithm was to automatically remove artifact without removing signal likely to be of neuronal origin from an ICA of resting-state fMRI. The algorithm assesses four types of artifacts; CSF, sparsely distributed noise, movement-related artifact, and high temporal frequency noise. The temporal feature of the IC is considered only after the initial clustering of ICs using spatial features because temporal frequency ranges of artifactual and neural activity sometimes overlap (Beckmann et al., 2005; Birn et al., 2006). Such overlap in frequencies complicates the determination of how much of component variance is due to artifacts vs. signal likely to be of neuronal origin.

We chose to use generic properties of the IC to categorize artifact, specifically limiting them to spatial features as we have particular interest in the application of SOCK to resting-state functional connectivity and to fMRI data collected prior to an epileptic seizure (Federico et al., 2005). In this case, we have no prior model of expected signal change or event timing (apart from the seizure itself at which point the data acquisition usually ends).

Despite the limited *a priori* information, SOCK was able to reduce the solution space by over 50% without rejecting any components that are likely to be of neuronal origin in our test group of 50 healthy controls. Only 0.3% (7) of components identified as artifact by SOCK were discordant with the manual classification and retrospective examination of these ICs suggested SOCK had correctly identified these as artifact. Thus, our method leads to a substantial reduction of the number of components to be inspected and interpreted.

There are other automatic classifier methods which like SOCK use a combination of spatial and temporal characteristics to inform IC classification (Thomas et al., 2002; Kochiyama et al., 2005; De Martino et al., 2007; Perlbarg et al., 2007; Tohka et al., 2008; Sui et al., 2009; Kundu et al., 2012), however, an important difference is that these methods either rely on training data (De Martino et al., 2007; Tohka et al., 2008) or task-related temporal or spatial information (Thomas et al., 2002; Kochiyama et al., 2005; Sui et al., 2009) or set thresholds (Perlbarg et al., 2007) or multi-echo acquisition sequences (Kundu et al., 2012). For example De Martino et al. (2007) used IC-fingerprints for characterizing independent components and in a support vector machines framework to classify them into six classes including activation and noise classes. However, the accuracy of their classifier was dependent on the training data. They found that automatic classification was less accurate in detecting residual motion signal effects due to small number of samples employed in the training. Other methods, such as Sui et al. (2009) classify ICs using contrast images that contain no time-domain information. Their method works on utilizing information concerning the proportion of active voxels overlapping ventricular CSF and gray-matter masks. From our experience, it is often difficult to obtain accurate gray-matter masks with EPI images at 3 T, as the boarders between GM and WM are often indistinct. Edge and CSF masks such as those used in SOCK can be more reliably extracted from EPI images than GM masks. The method of Sui et al. (2009) also requires a user-selected parameter (*Z*-score threshold) whereas SOCK uses the threshold automatically determined by the Gaussian mixture modeling approach implemented in MELODIC (Beckmann and Smith, 2004) when determining the edge and CSF activity and unthresholded maps when determining the degree of smoothness. Finally, the work of Kundu et al. (2012) offers a robust means for classifying components of interest vs. artifact based on TE-dependence. However, the applicability of this method is dependent on functional images being acquired with a multi-echo EPI sequence, which precludes it from use with data from studies not acquired in this fashion.

### 4.1 Limitations

The use of k-means clustering equips SOCK with objective and adaptive criteria, however it does require that at least some components are dominated by noise and others by signal of interest so that a meaningful clustering is achieved. This applies to clustering based upon degree of smoothness, edge activity, and temporal frequency noise. Even in the cases containing the fewest components in our study (subjects 25 and 27 in Table A2 in Appendix), SOCK was still effective at correctly removing a substantial proportion artifactual components: ICA yielded 14 components out of which SOCK classified 43 and 50% as artifact respectively. Nevertheless we advise caution in applying SOCK as presently implemented to variations of ICA that are already effective in producing very few, if any, components that are dominated by noise. For example event-related ICA (eICA) typically yields very few components due to it only dealing with short time epochs time-locked to events of interest (Masterton et al., 2013a,b). When only a handful of components are generated there is less need for an automatic classifier as it is relatively easy to manually inspect the ICs.

The performance of the SOCK classification is dependent on the accuracy of the edge and CSF masks which are generated using SPM’s New Segment tool. We found this tool robust in generating edge and CSF masks for the mean functional images used in this study. However, we did not test accuracy in cases where there exists gross pathology in subjects’ brains or where insufficient contrast between CSF and gray-matter regions exists in the EPI images. In these situations where the automated edge or CSF segmentation fails, the user could either generate custom masks, or elect to ignore these criteria (in which case SOCK would be unable to reject as artifact components exhibiting these features).

We tested the SOCK algorithm successfully on data from two different 3 T MRI scanners. These data had TR’s of 2.0, 3.0, and 3.6 s, voxel sizes of 2 mm × 2 mm × 2 mm and 3 mm × 3 mm × 3 mm and smoothing of 0 and 8 mm. The performance of SOCK was similar across all data sets. However, we only tested SOCK in conjunction with MELODIC (a popular ICA package). Other good ICA software exists [for example Egolf et al. (2004) and Himberg et al. (2004)] and we would therefore recommend a validation study if one were contemplating the use of SOCK with these or other packages.

A potential limitation of SOCK is that it may reject some neuronal activity if it is mixed with substantial noise in a single component. In the data we tested this may have occurred in two of the components we examined, as shown in Figure 8. In cases such as these it is not clear whether one should reject the component. One might argue that a conservative approach is rejection, because the potential activity of interest has the same time-course as noise. On the other hand, in clinical applications it may be considered conservative to retain the IC if any portion might be neuronal, even in the presence of noise. In the case of the two discordant components in this study, rejection would have had a minor impact on the possible neuronal activity, as there were other accepted components that contained substantially more activity in the same locations (see Figure 8).

In conclusion we have demonstrated a novel method for the automatic identification of artifactual ICs from resting-state fMRI data. SOCK proved to be effective in separating noise from signal in each of 50 healthy controls by identifying a high proportion of artifact-related ICs without removing components that are likely to be of neuronal origin. We tested the method with resting-state fMRI, however the method may also be effective for other study types and we therefore encourage validation studies in other contexts. SOCK does not require the user to train the algorithm and is able to adaptively determine variable threshold settings via use of k-means clustering. It does not require any temporal information about the fMRI paradigm or high-resolution anatomical scans. SOCK software is available at http://brain.org.au/software.

## 5 Conflict of Interest Statement

The authors declare that the research was conducted in the absence of any commercial or financial relationships that could be construed as a potential conflict of interest.

## Figures and Tables

**Figure A1:**
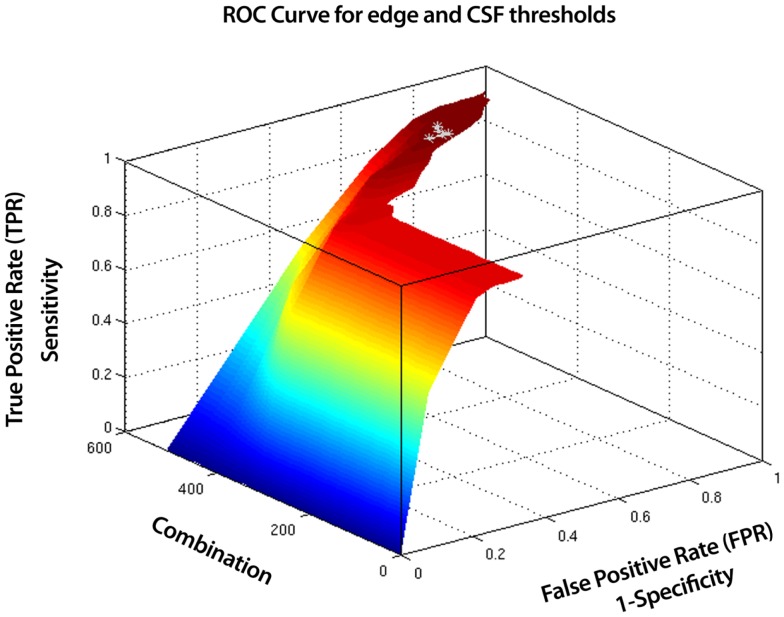
**Receiver Operating Characteristic (ROC) curve for edge and CSF thresholds for 5 test subjects**. A total of 525 combinations of edge and CSF thresholds (ranging from 0 to 70%) were tested. Shown in light gray are combinations where the classification in the 5 subjects did not change significantly (within 10%). These represent the ranges, 40–50% for the edge threshold and 20–30% for the CSF threshold. Lower values resulted in at least one neuronal component being misclassified as artifact (i.e., having a sensitivity of less than one which we regard as failure), whilst higher values resulted in fewer artifacts being identified (which we regard as a decrease in performance or smaller specificity). To minimize the chance of failure we chose the highest thresholds before a decrease in performance occurs (i.e., well away from the failure condition); 50 and 30% for the edge and CSF thresholds respectively.

**Figure A2:**
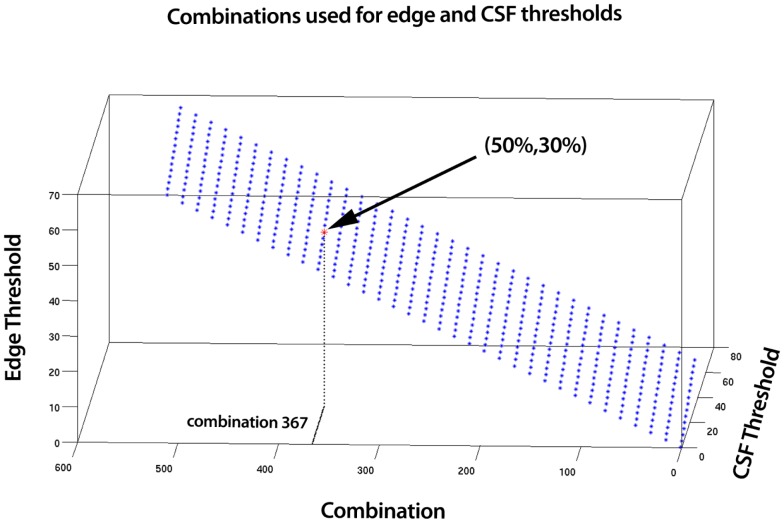
**A total of 525 combinations for edge and CSF thresholds ranging from 0 to 70% were tested**. Each combination represents an edge and CSF threshold, with combination 0 representing 0% edge and CSF threshold and combination 525 representing 70% edge and CSF threshold. Shown on the graph is combination 367, which corresponds to 50% edge threshold and 30% CSF threshold.

## Footnotes

^1^Temporal information expressed in the frequency domain. This is done mathematically by taking the discrete Fourier Transform of the time course.

^2^www.fil.ion.ucl.ac.uk/spm

^3^http://www.fil.ion.ucl.ac.uk/spm/software/spm8/

^4^www.brain.org.au/software

## Appendix A

**Table A1:** **ICA decomposition and the SOCK classification for 9 healthy controls who underwent a 60 min resting-state fMRI with TR of 3.0 s**.

Length of study (min)	TR (s)	Subject	Number of ICA components	SOCK classification		% of rejected ICs	No. of discordant ICs
	
				Artifact	Unlikely artifact	
60	3.0	1	34	17	17	50	1
60	3.0	2	34	19	15	56	0
60	3.0	3	127	60	67	47	0
60	3.0	4	87	44	43	51	0
60	3.0	5	97	54	43	56	1
60	3.0	6	84	33	51	39	0
60	3.0	7	108	55	53	51	0
60	3.0	8	129	76	53	59	3
60	3.0	9	58	41	17	71	0

*SOCK classified between 39 and 71% of each subject’s components as artifact (mean 53%). Only 5 of the components identified as artifact by SOCK were discordant with the manual classification (last column); retrospective examination of these ICs suggested SOCK had correctly identified these as artifact*.

**Table A2:** **ICA decomposition and the SOCK classification for 21 healthy controls who underwent a 10 min resting-state fMRI with TR of 3.6 s**.

Length of study (min)	TR (s)	Subject	Number of ICA components	SOCK classification		% of rejected ICs	No. of discordant ICs
	
				Artifact	Unlikely artifact	
10	3.6	10	22	9	13	41	0
10	3.6	11	22	12	10	55	0
10	3.6	12	24	11	13	46	0
10	3.6	13	21	12	9	57	0
10	3.6	14	21	10	11	48	0
10	3.6	15	26	15	11	58	0
10	3.6	16	19	6	13	32	0
10	3.6	17	21	8	13	38	0
10	3.6	18	22	11	11	50	0
10	3.6	19	22	12	10	55	0
10	3.6	20	18	8	10	44	0
10	3.6	21	17	6	11	35	0
10	3.6	22	19	5	14	26	0
10	3.6	23	16	9	7	56	0
10	3.6	24	19	11	8	58	0
10	3.6	25	14	6	8	43	0
10	3.6	26	18	7	11	39	0
10	3.6	27	14	7	7	50	0
10	3.6	28	18	11	7	61	0
10	3.6	29	16	6	10	38	0
10	3.6	30	21	11	10	52	0

*SOCK classified between 26 and 61% of each subject’s components as artifact (mean 47%). None of components identified as artifact by SOCK were discordant with the manual classification (last column)*.

**Table A3:** **ICA decomposition and the SOCK classification for 20 healthy controls who underwent a 9 min resting-state fMRI with TR of 2.0 s**.

Length of study (min)	TR (s)	Subject	Number of ICA components	SOCK classification		% of rejected ICs	No. of discordant ICs
	
				Artifact	Unlikely artifact	
9	2.0	31	79	50	29	63	0
9	2.0	32	100	49	51	49	0
9	2.0	33	78	41	37	53	0
9	2.0	34	74	45	29	61	0
9	2.0	35	72	40	32	56	0
9	2.0	36	109	54	55	50	0
9	2.0	37	82	59	23	72	0
9	2.0	38	63	41	22	65	0
9	2.0	39	78	45	33	58	0
9	2.0	40	58	33	25	57	0
9	2.0	41	79	50	29	63	2
9	2.0	42	98	57	41	58	0
9	2.0	43	90	56	34	62	0
9	2.0	44	57	26	31	46	0
9	2.0	45	83	51	32	61	0
9	2.0	46	79	51	28	65	0
9	2.0	47	78	54	24	69	0
9	2.0	48	63	32	31	51	0
9	2.0	49	74	43	31	58	0
9	2.0	50	60	25	35	42	0

*Data was obtained from the 1000 Functional Connectomes Project website. SOCK classified between 42 and 72% of each subject’s components as artifact (mean 58%). Only 2 of the components identified as artifact by SOCK were discordant with the manual classification (last column); retrospective examination of these ICs suggested SOCK had correctly identified these as artifact*.

## Appendix B

This appendix describes how we arrived at the hard thresholds used to determine when a component could be safely classified as artifact solely on edge or solely on CSF criteria. Our subsequent validation of the algorithm on data substantially different from that used here indicates that this procedure does not need to be re-done by an end user of the SOCK algorithm.

SOCK uses a combination of measures with adaptively determined thresholds to assist classification. However when there is a very large amount of edge or CSF activity, this alone can be enough to definitively classify the component as artifact. Therefore we use additional fixed thresholds to classify components as artifact when extremes occur in the edge and CSF measures. We tested a combination of fixed edge and CSF thresholds from 5 subjects scanned on the same scanner as data sets 1 and 2 in Section 2.5, where a manual classification was known. This was done via a Receiver Operating Characteristic (ROC) (Figure A1), which illustrates the performance of SOCK on these subjects for a total of 525 combinations of edge and CSF thresholds ranging from 0 to 70%. Each combination represents an edge and CSF threshold, with combination 0 representing 0% edge and CSF threshold and combination 525 representing 70% edge and CSF threshold (Figure A2). We found the classification in the 5 subjects did not change significantly (within 10%) in the range of 40–50% for edge threshold and 20–30% for the CSF threshold (points in light gray). Lower values resulted in at least one neuronal component being misclassified as artifact (i.e., having a sensitivity of less than one which we regard as failure), whilst higher values resulted in fewer artifacts being identified (which we regard as a decrease in performance or smaller specificity). To minimize the chance of failure we chose the highest thresholds before a decrease in performance occurs (i.e., well away from the failure condition); 50 and 30% for the edge and CSF thresholds respectively.
